# Social Vulnerability Predictors of Acute Care: Leveraging Health Information Exchange Data to Understand Social Determinants at the Census Tract Level in a Correlational Study

**DOI:** 10.1002/hsr2.71257

**Published:** 2025-09-29

**Authors:** Karen L. Pellegrin, Tanner B. Barbour, Alicia J. Lozano, Alexandra L. Hanlon

**Affiliations:** ^1^ University of Hawaiʻi at Hilo Hilo Hawaiʻi USA; ^2^ Virgina Tech Blacksburg Virginia USA

**Keywords:** acute care utilization, census tracts, emergency department, Hawaiʻi, health information exchange, inpatient, rural, social vulnerability

## Abstract

**Background and Aims:**

While geographic social vulnerability is a known predictor of acute care utilization, it is not known which specific vulnerabilities are the best predictors. This is particularly important in rural areas where there are significant disparities. The purpose of this study was to identify social vulnerability predictors of acute care utilization across and within rural counties in Hawaiʻi.

**Methods:**

This correlational study examined counts of emergency department (ED) visits and inpatient (IP) admissions for any reason by census tract obtained from Hawaiʻi Health Information Exchange for rural counties in Hawaiʻi. The overall Social Vulnerability Index (SVI), SVI subthemes, and individual measures that comprise the composites were used as measures of social vulnerability for each census tract. Regression models analyzed counts per population, after adjustments for missing data, where the response variable represents the number of events occurring. Each outcome (number of ED or IP visits) was regressed on a single predictor of social vulnerability for each county and for all counties combined.

**Results:**

Across counties, the largest significant effect associated with acute care utilization was overall social vulnerability (ED: IRR = 5.72, 95% CI = 5.55–5.89; IP: IRR = 5.76, 95% CI = 5.42–6.12). The largest effect within Kauaʻi County was Racial and Ethnic Minority Status (ED: IRR = 5.38, 95% CI = 5.13–5.64; IP: IRR = 6.30, 95% CI = 5.64–7.03), within Maui County was Housing Type and Transportation (ED: IRR = 6.72, 95% CI = 6.37–7.1; IP: IRR = 4.46, 95% CI = 3.99–5), and within Hawaiʻi County was Household Characteristics for ED (IRR = 11.50, 95% CI = 10.91–12.12) and No High School Diploma for IP (IRR = 6.33, 95% CI = 5.79–6.93).

**Conclusions:**

Social vulnerability is a significant predictor of acute care utilization across rural areas in Hawaiʻi. The strongest predictors were different for each county.

## Summary

- What is known on this topic?
  ◦ Higher social vulnerability, both at the county level and census tract level, predicts greater acute care use and other health disparities.
  ◦ County‐level social vulnerability indicators, specifically median income and percent of the population in poverty, explain rural disparities in premature mortality across the USA.
  ◦ Rural areas have higher rates of ED visits for which patients are treated and released relative to metropolitan areas after excluding those visits associated with a mental health or substance use disorder.

- What this study adds?
  ◦ The statewide Hawaiʻi Health Information Exchange provided population counts of ED visits and inpatient admissions by census tract for the three rural counties in Hawaiʻi.
  ◦ Across rural counties in Hawaiʻi, the CDC's overall social vulnerability index was the best predictor of acute care utilization by census tract relative to the CDC's vulnerability themes or individual measures.
  ◦ Within each rural county, the best social vulnerability predictor of acute care utilization by census tract was not the overall index and was different for each county, indicating the importance of more granular analyses to guide local health equity efforts.

## 1 Introduction

Two county‐level indicators of socioeconomic vulnerability alone—median income and percent of the population in poverty—have been shown to explain 50% of the variation in age‐adjusted premature mortality across counties in the United States (US), and after adjusting for these social determinants, rural–urban differences in mortality largely disappear [1]. Composite social vulnerability measures provide a tool to support planning and decision‐making by combining multiple indicators of vulnerability at the community level. The CDC's Social Vulnerability Index (SVI) is an easily accessible composite measure which also readily allows examination of the individual measures that comprise the composite index.

While there have been criticisms of this SVI measure, the concerns raised have not invalidated use of the SVI for understanding comparative social vulnerabilities to support decision‐making and have inspired further research [2]. For example, research indicates that the SVI more accurately classifies at least some census tracts compared to the Area Deprivation Index [3] and has better discriminative validity relative to the Area Deprivation Index [4]. Although the SVI was originally developed to help officials better prepare for and respond to disasters by identifying more vulnerable communities, its value has rapidly translated to use in the healthcare industry more broadly and specifically to study and address health disparities.

The SVI developed by the Centers for Disease Control (CDC) is a composite indicator composed of 16 US census variables and has been used to study geographic health disparities [5]. The SVI measures are available at the county and subcounty level. County‐level social vulnerability has been found to predict acute care use and other health outcomes, including critical intensive care unit capacity during the pandemic [6], inpatient mortality rates among those hospitalized with COVID‐19 [7], and trauma fatality rates [8]. In addition, an analysis of data from 33 states indicates that county‐level preventable hospitalization rates were 40% higher in counties with the highest social vulnerability relative to counties with the lowest vulnerability [9].

### 1.1 Research on Census Tract‐Level Social Vulnerability

Social vulnerability research at the census tract‐level provides a more granular view to assessing community health needs. In a cross‐sectional study of nearly 900,000 Medicare beneficiaries who had emergency general surgery, those living in census tracts within the highest quintile of social vulnerability had higher rates of 30‐day mortality, serious complications, and readmissions compared to those living in tracts within the lowest quintile, after adjusting for demographics, comorbidity, type of surgery, and hospital characteristics [10]. In a study of more than 3000 pediatric surgeries, those patients at or above the 90th percentile on neighborhood social vulnerability had worse surgical outcomes after controlling for demographics, insurance status, and language preference [11]. In another study of more than 37,000 pediatric head and neck cancer patients, those living in a neighborhood with higher social vulnerability were less likely to receive care, such as surgery, and had decreased survival relative to those with lower vulnerability [12]. Another study found that, compared to counties in the lowest quintile of social vulnerability, those with higher vulnerability had higher rates of asthma‐related emergency department (ED) visits and hospitalizations [13].

The role of social vulnerability in acute care utilization is important not only to address health disparities but also to address the sustainability of the US healthcare system as hospital care is the single largest category of healthcare expenditures [14]. In response to concerns about potential overuse or inappropriate use of EDs, the Office of the Assistant Secretary for Planning and Evaluation, US Department of Health & Human Services used data from the Agency for Healthcare Research and Quality Healthcare Cost and Utilization Project to examine trends in ED utilization from 2009 to 2018. In their report to Congress [15], they included analyses of those ED visits leading to hospital admission versus treated and released and those associated with mental health or substance use disorder (MH/SUD) diagnoses versus those without one or more of these diagnoses. The overall annual ED use rates ranged from approximately 42,000 to 45,000 ED visits per 100,000, and the large majority of these visits were not associated with a MH/SUD diagnosis and did not result in hospital admission. In 2018, among those ED visits not associated with a MH/SUD diagnosis but that resulted in hospital admission, the ED use rate varied substantially by age. Among those under age 45, the ED visit rate was under 2000/100,000. Among those age 45–64, it was ~3600/100,000. Among those age 65 and older, it was more than 13,000/100,000. In a comparison by geography, focusing on those ED visits not associated with a MH/SUD diagnosis, rural areas had lower rates of ED visits that resulted in hospitalization and higher rates of ED visits for which patients were treated and released relative to metropolitan areas. This may be an indicator of rural populations with less access to primary care using EDs as a substitute. They also found a consistent pattern related to levels of social vulnerability. Across all categories of ED visits—those that resulted in admission, those that were treated and released, and those associated with MH/SUD diagnosis—the ED utilization rates were significantly higher in census tracts that were in the highest quartile of social vulnerability relative to the lowest quartile [15].

### 1.2 Research Gaps

The links between social vulnerabilities and acute care utilization at the census tract‐level *within* counties to support improvements in health equity have not been well studied. In particular, there is a paucity of information about the specific social vulnerabilities that are associated with acute care use in rural communities and whether these specific vulnerabilities are the same across rural areas. Understanding the specific vulnerabilities that impact acute care utilization may lead to more effective solutions within local rural communities where there are significant health disparities.

The CDC's focus on geographic health disparities via social vulnerability measures provides potential opportunities to leverage data captured through state or regional health information exchange (HIE) systems. Specifically, incorporating social determinant data into HIEs is recognized as a promising approach to identifying needs and tracking community health outcomes [16]. Hawaiʻi Health Information Exchange (HHIE) is the state‐designated entity for the exchange of health information in Hawaiʻi and can identify the census tract where a patient lives based on home address, which allows census tract‐level social determinants to be incorporated. Thus, the purpose of this study is to explore social vulnerability predictors of acute care utilization using census tract‐level data across and within rural counties in Hawaiʻi. With this exploratory study, our working hypotheses were that (1) social vulnerability measures would be significantly associated with acute care utilization rates and (2) the specific vulnerabilities most strongly associated with acute care utilization rates may vary by county.

## 2 Methods

This study used a correlational design to assess the relationship between acute care utilization and social vulnerability at the census tract level within the three most rural counties in Hawaiʻi based on the CDC's National Center for Health Statistics Urban–Rural Classification Scheme for Counties. This scheme classifies counties using a six‐level scale, with 1 being the *most urban* and 6 the *most rural*. In Hawaiʻi, Honolulu County is the most urban and classified as 3, and the other three counties are classified as 4 or 5 [17]. The difference between Honolulu and the other counties is seen more clearly in resident population density; Honolulu County has 1692 residents per square mile, while Maui County has 140, Kauaʻi has 118, and Hawaiʻi County (aka “Big Island”) has 50 residents per square mile [18]. While Honolulu County has the highest overall social vulnerability based on the CDC's SVI composite measure at the county level, it has the lowest vulnerability based on the CDC's socioeconomic theme. Hawaiʻi County has the highest socioeconomic vulnerability, followed by Kauaʻi and then Maui counties [5]. Within the rural counties, Maui has 44 census tracts, Kauaʻi has 17, and Hawaiʻi has 43.

### 2.1 Data Source

The number of inpatient (IP) admissions and ED visits for any reason from April 1, 2022 to March 31, 2023 by census tract in the three rural counties in Hawaiʻi (i.e., Hawaiʻi County also known as the “Big Island,” Kauaʻi County, and Maui County) were obtained from HHIE, after they mapped patient home addresses from the encounters to the census tract. HHIE receives ED and IP admissions data from all acute care facilities in those three counties and all but one nonfederal general acute care facilities in Honolulu County. Only those encounters with a patient home address that matched exactly to a census tract were included in the data from HHIE. Those addresses that did not match exactly to a census tract were categorized by county using the zip code so that percent match rates could be compared by county.

### 2.2 Measures

**Figure 1:**
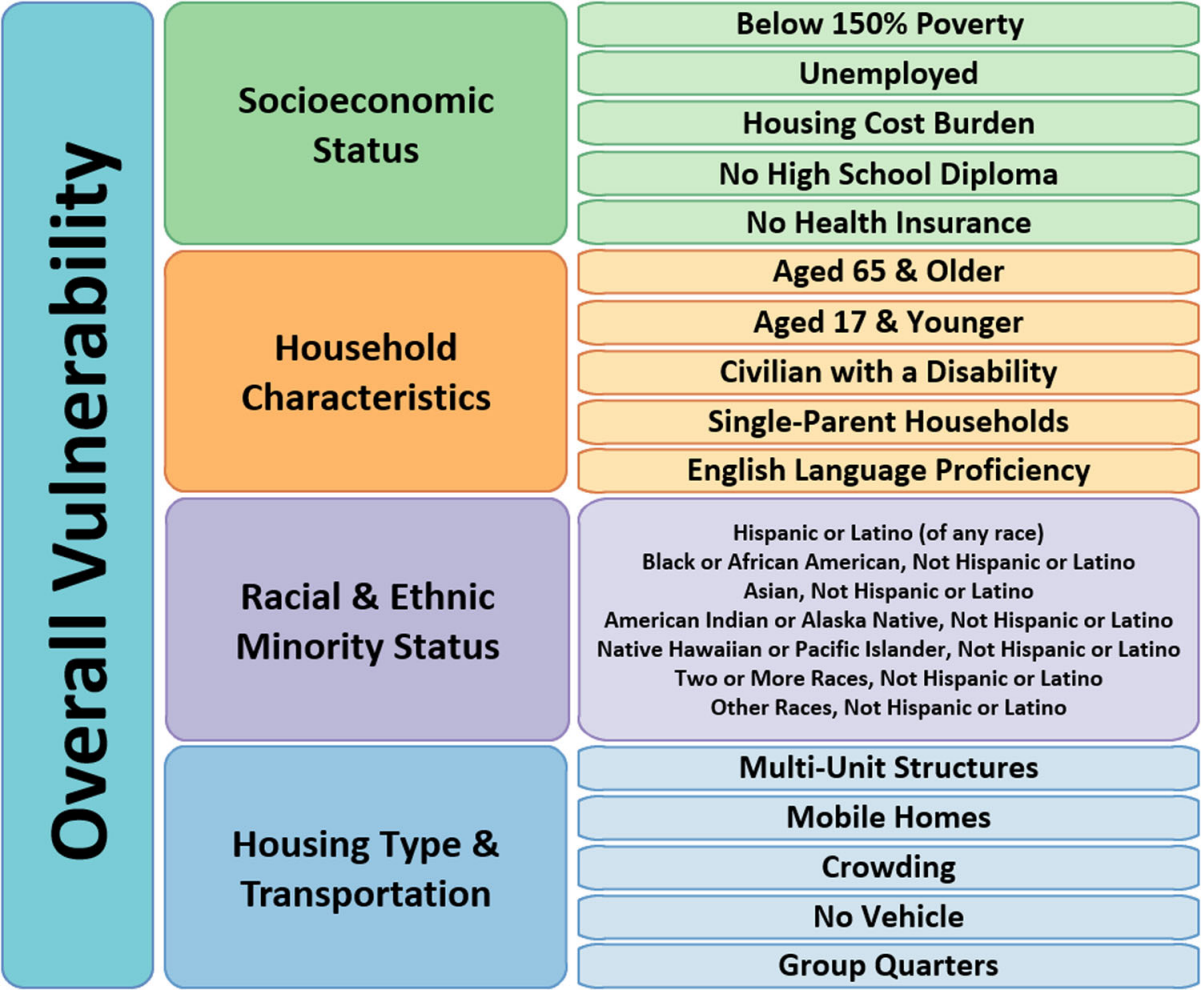
CDC's social vulnerability measures and composite measures.

The outcomes of interest were counts of IP admissions and ED visits for each census tract from April 1, 2022 to March 31, 2023. Census tract‐level population was used to create rates (count per population). Within each county, the census tract rates were adjusted based on the percent of missing data due to unmatched patient addresses for the respective county. The CDC's 2020 SVI ranks by census tract were used across rural counties in Hawaiʻi. This included census tract ranks for all 16 social factors that the CDC uses to identify populations vulnerable to major health and social threats. This also included the census tract ranks for the four composite SVI themes (Socioeconomic Status, Household Composition, Racial and Ethnic Minority Status, and Housing Type and Transportation) and the ranks for the composite overall SVI [19]. Figure 1 shows how the 16 measures map to the composite themes and overall index measures [19].

The CDC's census tract‐level social vulnerability measures were used as the predictors of interest, including the overall score, themes, and individual measures. These measures range from 0 to 1, with higher scores reflecting a more vulnerable community. All 16 measures that comprise the overall SVI were included, along with the overall SVI and each of the themes.

### 2.3 Statistical Analysis

Descriptive statistics for SVI scores and admissions in each county were performed. Normality was assessed using the Shapiro–Wilk test, where means and standard deviations (SDs) were used for normally distributed data, and medians and ranges or interquartile ranges (IQRs) for data that were not normally distributed. Across the three counties in aggregate and within each county, generalized linear models specifying a Poisson distribution at the census tract level were performed both in aggregate across the three counties and within each county. Poisson models are appropriate to analyze count outcome data. In these bivariate analyses, each outcome (number of ED or inpatient visits per 1000 population) was regressed on a single predictor variable of interest (overall SVI, SVI themes, and SVI subthemes, separately; see Section 2.2 for more details). Incidence rate ratios (IRRs), which express the ratio of the incidence rate of an event (e.g., admission, disease, injury, death, etc.), in one group compared to another over a specified period of time [20], and their 95% confidence intervals (CI) were calculated and reported for each model. Statistical significance was taken at the 0.05 level and did not account for multiplicity. All analyses were two‐sided, prespecified, and performed in R Statistical Software (v4.3.1; R Core Team 2023).

## 3 Results

Table 1 shows the counts aggregated across all three counties (“Total” column) as well as by county. The total number of ED visits and IP admissions with addresses that mapped to a census tract (unadjusted utilization), the number and percent of addresses from any encounter that matched a census tract address, and the utilization rates after adjusting for the county‐level match rate by rural county in Hawaiʻi, dates from April 1, 2022 to March 31, 2023.

**Table 1:** Unadjusted and adjusted acute care utilization and social vulnerability measures (county level).

	Total	Hawaiʻi	Maui	Kauaʻi
Unadjusted utilization				
Count of ED visits	68,077	30,003	20,020	18,054
Count of IP visits	14,871	7216	4373	3282
Adjusted utilization				
Count of ED visits	87,936	41,671	25,025	21,240
Count of IP visits	19,349	10,022	5466	3861
Unique addresses				
Total addresses	207,383	117,744	50,394	39,245
Matched addresses	158,295	84,936	40,106	33,253
Matched addresses match rate ((Matched Addresses/Total Addresses) * 100)	76%	72%	80%	85%
Unmatched addresses	49,088	32,808	10,288	5992
Unmatched addresses match rate ((Unmatched Addresses/Total Addresses) * 100)	24%	28%	20%	15%
Total population				
US Census 5‐year estimate, 2020	439,956	201,350	166,657	71,949
Unadjusted acute care utilization rates				
ED visits per 1000	155	149	120	251
IP visits per 1000	34	36	26	46
Acute care utilization ADJUSTED for missing data due to unmatched address				
ED visits per 1000		207	150	295
IP visits per 1000		50	33	54

SVI variable	Total	Hawaiʻi	Maui	Kauaʻi
*Socioeconomic*	0.67 (0.45–0.83)^a^	0.74 (0.42–0.87)^a^	0.65 (0.49–0.78)^a^	0.60 (0.17)^b^
Below 150 Percentage Poverty	0.67 (0.43–0.81)^a^	0.80 (0.57–0.90)^a^	0.54 (0.26)^b^	0.61 (0.16)^b^
Unemployed	0.62 (0.36–0.83)^a^	0.73 (0.37–0.89)^a^	0.60 (0.26)^b^	0.45 (0.22)^b^
Housing Cost Burden	0.53 (0.20)^b^	0.53 (0.17)^b^	0.52 (0.24)^b^	0.54 (0.16)^b^
No High School Diploma	0.54 (0.34–0.77)^a^	0.60 (0.37–0.74)^a^	0.54 (0.26)^b^	0.53 (0.27)^b^
No Health Insurance	0.69 (0.47–0.87)^a^	0.62 (0.26)^b^	0.73 (0.48–0.88)^a^	0.74 (0.61–0.82)^a^
*Household Characteristics*	0.57 (0.33–0.78)^a^	0.75 (0.58–0.89)^a^	0.42 (0.26)^b^	0.49 (0.25)^b^
Age 65 and Older	0.54 (0.36–0.74)^a^	0.60 (0.20)^b^	0.42 (0.28–0.72)^a^	0.60 (0.19)^b^
Aged 17 and Younger	0.60 (0.35–0.75)^a^	0.56 (0.23)^b^	0.59 (0.31–0.76)^a^	0.63 (0.18)^b^
Civilian with a Disability	0.48 (0.24–0.76)^a^	0.76 (0.50–0.92)^a^	0.28 (0.17–0.51)^a^	0.36 (0.17)^b^
Single‐Parent Households	0.60 (0.37–0.77)^a^	0.64 (0.39–0.74)^a^	0.55 (0.28–0.83)^a^	0.48 (0.24)^b^
English Language Proficiency	0.46 (0.23–0.66)^a^	0.49 (0.21)^b^	0.35 (0.16–0.60)^a^	0.44 (0.27)^b^
*Racial and Ethnic Minority Status*	0.27 (0.15–0.48)^a^	0.28 (0.18–0.42)^a^	0.28 (0.10–0.48)^a^	0.32 (0.25)^b^
Minority	0.27 (0.15–0.48)^a^	0.28 (0.18–0.42)^a^	0.28 (0.10–0.48)^a^	0.32 (0.25)^b^
*Housing Type and Transportation*	0.53 (0.31–0.70)^a^	0.44 (0.27–0.70)^a^	0.54 (0.24)^b^	0.50 (0.21)^b^
Multiunit Structures	0.44 (0.25–0.66)^a^	0.36 (0.00–0.58)^a^	0.58 (0.38–0.68)^a^	0.46 (0.26)^b^
Mobile Homes	0.00 (0.00–0.83)^a^	0.00 (0.00–0.84)^a^	0.00 (0.00–0.80)^a^	0.00 (0.00–0.86)^a^
Crowding	0.52 (0.24)^b^	0.48 (0.24)^b^	0.57 (0.25)^b^	0.51 (0.22)^b^
No Vehicle	0.44 (0.23)^b^	0.45 (0.24)^b^	0.42 (0.24)^b^	0.45 (0.18)^b^
Group Quarters	0.46 (0.00–0.68)^a^	0.50 (0.00–0.69)^a^	0.46 (0.00–0.62)^a^	0.50 (0.00–0.72)^a^
*Overall Vulnerability*	0.56 (0.22)^b^	0.62 (0.22)^b^	0.52 (0.23)^b^	0.54 (0.18)^b^

Abbreviations: IQR, interquartile range (1st quartile–3rd quartile); SD, standard deviation.

^a^ Median (IQR).

^b^ Mean (SD).

Across the three rural counties as shown in Table 2, all but one social vulnerability measure had a significant effect associated with acute care utilization, and the largest effect was the overall index (ED: IRR = 5.72, 95% CI = 5.55–5.89, *p* < 0.001; IP: IRR = 5.76, 95% CI = 5.42–6.12, *p* < 0.001). Specifically, for each 1‐point increase in the overall social vulnerability measure, the number of ED visits increases 5.7‐fold and the number of IP admissions increases 5.8‐fold. That is, more social vulnerability is associated with more ED visits and IP admissions across all rural counties.

**Table 2:** Predictors of acute care utilization rates across all counties.

		Adjusted acute care utilization rates					
		ED			IP		
Model	Predictor variable	IRR	95% CI	*p*	IRR	95% CI	*p*
1	*Socioeconomic Status*	3.50	3.4–3.6	< 0.001	2.75	2.59–2.92	< 0.001
2	Below 150% Poverty	3.74	3.64–3.85	< 0.001	2.81	2.66–2.98	< 0.001
3	Unemployed	1.10	1.08–1.13	< 0.001	0.70	0.67–0.73	< 0.001
4	Housing Cost Burden	1.16	1.13–1.2	< 0.001	1.06	1–1.13	0.06
5	No High School Diploma	3.79	3.69–3.89	< 0.001	3.88	3.67–4.1	< 0.001
6	No Health Insurance	1.72	1.68–1.76	< 0.001	2.06	1.96–2.17	< 0.001
7	*Household Characteristics*	3.02	2.95–3.09	< 0.001	3.35	3.19–3.51	< 0.001
8	Age 65 & Older	0.66	0.65–0.68	< 0.001	0.82	0.78–0.87	< 0.001
9	Aged 17 & Younger	2.45	2.39–2.52	< 0.001	1.87	1.77–1.98	< 0.001
10	Civilian with a Disability	2.11	2.06–2.15	< 0.001	2.01	1.93–2.1	< 0.001
11	Single‐Parent Households	1.61	1.57–1.64	< 0.001	1.17	1.12–1.23	< 0.001
12	English Language Proficiency	1.85	1.81–1.9	< 0.001	3.39	3.22–3.57	< 0.001
13	*Racial and Ethnic Minority Status*	4.47	4.36–4.59	< 0.001	2.80	2.66–2.95	< 0.001
14	Minority	4.47	4.36–4.59	< 0.001	2.80	2.66–2.95	< 0.001
15	*Housing Type and Transportation*	2.08	2.03–2.13	< 0.001	2.79	2.64–2.94	< 0.001
16	Multiunit Structures	0.79	0.78–0.81	< 0.001	1.12	1.07–1.17	< 0.001
17	Mobile Homes	1.01	1–1.03	0.08	0.95	0.93–0.98	< 0.001
18	Crowding	1.34	1.3–1.37	< 0.001	1.76	1.66–1.85	< 0.001
19	No Vehicle	4.14	4.03–4.25	< 0.001	3.57	3.37–3.78	< 0.001
20	Group Quarters	1.93	1.9–1.97	< 0.001	2.31	2.23–2.4	< 0.001
21	*Overall Vulnerability*	5.72	5.55–5.89	< 0.001	5.76	5.42–6.12	< 0.001

*Note:* The green‐shaded p's indicate statistical significance.

Abbreviations: CI, confidence interval; ED, emergency department; IP, inpatient admissions; IRR, incidence rate ratios.

Table 3 shows the results by county. The largest effect within Kauaʻi County was Racial & Ethnic Minority Status (ED: IRR = 5.38, 95% CI = 5.13–5.64, *p* < 0.001; IP: RR = 6.30, 95% CI = 5.64–7.03, *p* < 0.001). Specifically, for each 1‐point increase in the Racial & Ethnic Minority measure in Kauaʻi County, the number of ED visits increases 5.4‐fold and the number of IP admissions increases 6.3‐fold.

**Table 3:** Predictors of acute care utilization rates by county.

		Adjusted acute care utilization rates																	
		Hawaiʻi county						Maui county						Kauaʻi county					
		ED			IP			ED			IP			ED			IP		
Model	Predictor variable	IRR	95% CI	*p*	IRR	95% CI	*p*	IRR	95% CI	*p*	IRR	95% CI	*p*	IRR	95% CI	*p*	IRR	95% CI	*p*
1	*Socioeconomic Status*	4.69	4.5–4.88	< 0.001	2.24	2.08–2.41	< 0.001	2.41	2.28–2.54	< 0.001	2.18	1.93–2.45	< 0.001	2.58	2.4–2.77	< 0.001	3.51	2.96–4.16	< 0.001
2	Below 150% Poverty	10.38	9.86–10.93	< 0.001	2.04	1.87–2.22	< 0.001	1.32	1.26–1.38	< 0.001	1.43	1.29–1.58	< 0.001	2.26	2.09–2.44	< 0.001	3.26	2.72–3.9	< 0.001
3	Unemployed	2.63	2.55–2.72	< 0.001	0.75	0.71–0.8	< 0.001	0.75	0.72–0.79	< 0.001	0.81	0.73–0.89	< 0.001	0.46	0.44–0.48	< 0.001	0.53	0.47–0.61	< 0.001
4	Housing Cost Burden	0.99	0.94–1.04	0.69	0.80	0.72–0.88	< 0.001	1.45	1.38–1.52	< 0.001	1.30	1.17–1.44	< 0.001	0.83	0.77–0.9	< 0.001	0.97	0.81–1.16	0.75
5	No High School Diploma	5.57	5.32–5.84	< 0.001	6.33	5.79–6.93	< 0.001	3.37	3.21–3.53	< 0.001	2.63	2.38–2.91	< 0.001	2.88	2.75–3.02	< 0.001	2.96	2.66–3.31	< 0.001
6	No Health Insurance	1.84	1.78–1.91	< 0.001	2.83	2.64–3.03	< 0.001	1.42	1.36–1.48	< 0.001	1.40	1.28–1.54	< 0.001	1.64	1.55–1.73	< 0.001	1.75	1.53–2	< 0.001
7	*Household Characteristics*	11.50	10.91–12.12	< 0.001	4.54	4.14–4.98	< 0.001	3.09	2.96–3.23	< 0.001	2.44	2.22–2.68	< 0.001	0.71	0.68–0.75	< 0.001	0.95	0.85–1.06	0.34
8	Age 65 & Older	0.39	0.38–0.41	< 0.001	0.54	0.49–0.58	< 0.001	0.65	0.62–0.68	< 0.001	0.72	0.65–0.8	< 0.001	0.17	0.16–0.19	< 0.001	0.18	0.15–0.21	< 0.001
9	Aged 17 & Younger	3.09	2.96–3.22	< 0.001	1.77	1.64–1.92	< 0.001	2.37	2.26–2.49	< 0.001	2.03	1.83–2.25	< 0.001	0.73	0.68–0.78	< 0.001	0.99	0.85–1.17	0.95
10	Civilian with a Disability	7.12	6.82–7.43	< 0.001	1.93	1.8–2.08	< 0.001	1.00	0.95–1.04	0.87	0.97	0.88–1.07	0.52	0.43	0.4–0.47	< 0.001	0.46	0.38–0.54	< 0.001
11	Single‐Parent Households	3.10	2.98–3.23	< 0.001	1.10	1.03–1.18	0.008	1.53	1.47–1.59	< 0.001	1.28	1.18–1.39	< 0.001	1.03	0.97–1.08	0.35	1.06	0.94–1.2	0.32
12	English Language Proficiency	0.82	0.79–0.86	< 0.001	3.95	3.63–4.3	< 0.001	2.72	2.6–2.84	< 0.001	2.05	1.87–2.25	< 0.001	1.83	1.75–1.92	< 0.001	2.43	2.18–2.7	< 0.001
13	*Racial and Ethnic Minority Status*	3.89	3.73–4.05	< 0.001	1.41	1.29–1.53	< 0.001	4.74	4.53–4.95	< 0.001	3.94	3.59–4.34	< 0.001	5.38	5.13–5.64	< 0.001	6.30	5.64–7.03	< 0.001
14	Minority	3.89	3.73–4.05	< 0.001	1.41	1.29–1.53	< 0.001	4.74	4.53–4.95	< 0.001	3.94	3.59–4.34	< 0.001	5.38	5.13–5.64	< 0.001	6.30	5.64–7.03	< 0.001
15	*Housing Type and Transportation*	1.63	1.58–1.69	< 0.001	3.37	3.15–3.6	< 0.001	6.72	6.37–7.1	< 0.001	4.46	3.99–5	< 0.001	1.86	1.76–1.97	< 0.001	1.50	1.32–1.71	< 0.001
16	Multiunit Structures	0.96	0.93–0.99	0.01	2.35	2.21–2.51	< 0.001	0.94	0.9–0.98	< 0.001	0.84	0.76–0.93	0.001	1.05	1–1.11	0.035	1.16	1.04–1.3	< 0.001
17	Mobile Homes	0.81	0.79–0.83	< 0.001	0.80	0.77–0.84	< 0.001	2.14	2.08–2.2	< 0.001	1.91	1.8–2.02	< 0.001	0.60	0.58–0.62	< 0.001	0.51	0.48–0.55	< 0.001
18	Crowding	1.41	1.36–1.46	< 0.001	2.60	2.42–2.79	< 0.001	2.06	1.96–2.16	< 0.001	1.88	1.69–2.09	< 0.001	1.96	1.86–2.08	< 0.001	2.50	2.19–2.85	< 0.001
19	No Vehicle	5.20	4.99–5.42	< 0.001	4.14	3.82–4.48	< 0.001	4.46	4.24–4.69	< 0.001	3.65	3.28–4.07	< 0.001	1.58	1.48–1.68	< 0.001	1.18	1.02–1.37	0.024
20	Group Quarters	1.32	1.29–1.35	< 0.001	2.15	2.04–2.27	< 0.001	2.44	2.36–2.53	< 0.001	1.98	1.84–2.14	< 0.001	2.74	2.65–2.84	< 0.001	2.59	2.4–2.8	< 0.001
21	*Overall Vulnerability*	7.79	7.43–8.15	< 0.001	5.77	5.29–6.29	< 0.001	5.84	5.54–6.17	< 0.001	4.10	3.66–4.59	< 0.001	2.98	2.78–3.18	< 0.001	3.70	3.16–4.33	< 0.001

*Note:* The green‐shaded p's indicate statistical significance.

The largest effect within Maui County was Housing Type and Transportation (ED: IRR = 6.72, 95% CI = 6.37–7.1, *p* < 0.001; IP: IRR = 4.46, 95% CI = 3.99–5, *p* < 0.001). Specifically, for each 1‐point increase in the Housing Type and Transportation measure in Maui County, the number of ED visits increases 6.7‐fold and the number of IP admissions increases 4.5‐fold.

The largest effect within Hawaiʻi County was Household Characteristics for ED (IRR = 11.50, 95% CI = 10.91–12.12, *p* < 0.001) and No High School Diploma for IP (IRR = 6.33, 95% CI = 5.79–6.93, *p* < 0.001). Specifically, in Hawaiʻi County, for each 1‐point increase in the Household Characteristics measure, the number of ED visits increases 11.5‐fold, and for each 1‐point increase in the No High School Diploma measure, the number of IP admissions increases 6.3‐fold.

## 4 Discussion

The CDC's social vulnerability measures are significant predictors of acute care utilization in rural Hawaiʻi. However, the specific measures with the largest effects differed by county, suggesting the importance of examining data at a more granular census tract level within counties to guide local efforts to improve health equity. The largest effects were seen within Hawaiʻi County, a finding which may be related to it having the highest overall SVI at the county level, relative to the other rural counties in the state. Hawaiʻi County is also the most rural, based on population density, and has the highest unemployment and highest poverty relative to the other rural counties and urban Honolulu County. It is unclear why the best predictor of IP utilization was different from the best predictor of ED utilization in Hawaiʻi County. It is possible that ED utilization may be affected to a greater extent than IP utilization by socioeconomic conditions such as houselessness and food insecurity.

In Hawaiʻi County, not having a high school diploma was the best predictor of IP utilization, suggesting opportunities to focus health literacy efforts in census tracts with lower high school graduation rates. The Household Characteristics theme, and particularly the noninstitutionalized civilian population with a disability measure within that theme, was the best predictor of ED utilization in Hawaiʻi County. The US Census Bureau has noted that disability rates are higher in rural areas across the US relative to urban areas and the importance of ensuring specialized services are available to address disabilities [21]. This could include, for example, transportation services and housing units designed to support improved safety, ability to live independently, and access to care in census tracts with a greater proportion of residents with a disability. Our research indicates the potential for such services to be cost‐effective as they may reduce acute care utilization.

Across counties, one of the weaker predictors of acute care utilization was the population age 65 and older measure. This is particularly surprising for IP utilization given that this age group is more than twice as likely to have one or more hospital admissions each year [22]. In addition, previous research in Hawaiʻi found that the county with the highest percent of the population age 65+ (Kauaʻi County, 14.9%) also had the highest percent of age 65+ hospital admissions for any reason (33.8%), while the county with the lowest percent of the population age 65+ (Maui County, 12.8%) had the lowest percent of age 65+ hospital admissions for any reason (26.7%) [23]. This previous research puts into context our current findings, underscoring the greater impact of other social determinants of health relative to age in explaining variation in acute care utilization. Additional research is needed to understand the potential interactions between these social determinants and their effects on acute care utilization.

A limitation of this study is the missing data due to the inability to map some patient encounters to a census tract. This mapping process is secondary to the primary purpose of HIEs, which is to aggregate a patient's electronic health data from across providers to support clinical decision making at the point of care. Thus, the rates of acute care use at the census tract level are only based on available data, potentially introducing bias. While the census tract‐level data were adjusted based on the percent of patient addresses missing for the county where the census tract is located, this approach would not accurately adjust the data if there are large differences in missing data among the census tracts within a county. Furthermore, any such large differences within or across counties may introduce bias. For example, according to the US Census Bureau, the reasons addresses might not map to a census tract or other geography include recently constructed housing units that are not yet in the census database and, although rare, sparsely populated areas [24], the latter of which would disproportionately underestimate counts in the most rural census tracts. Other reasons may include incomplete or inaccurate information in the patient's health record, though how these errors would affect the results is unclear. Future research could adjust for missing data at a more granular level by using the zip code‐level address match rate to adjust for the missing data at the census tract level and perform regression analyses using these adjusted counts.

The one nonfederal general acute care hospital located in Honolulu County that does not transmit ED or IP admission data to HHIE, Kaiser Permanente Moanalua Medical Center, is another source of missing data. This hospital accounts for 11% of nonfederal, short‐term, acute care IP admissions in the state [25]. These missing data are more likely to affect IP than ED rates because Kaiser members who reside in the three rural counties would visit a local ED for emergency care but may travel to Honolulu County for IP care. Another limitation of this study is that conclusions about causality cannot be confirmed given the correlational design. While it is unlikely that acute care use causes social vulnerabilities, it is possible that unmeasured mediating variables are the causal factors rather than the specific vulnerabilities included in the measures for this study. Future research should also include more urban counties to examine potential differences in social vulnerability predictors in these areas. Finally, while this study included all acute care utilization, additional research is needed to determine if predictors vary by health condition.

Despite these limitations, state, local, and regional HIEs can be leveraged to support health equity efforts by incorporating social determinants of health. The CDC's SVI, themes, and individual measures that comprise the composite measures are strong predictors of acute care utilization in rural Hawaiʻi. The specific predictors that were the strongest were different within each county, demonstrating the value of more granular analyses at the census tract level to guide planning and local allocation of resources to address disparities within counties.

## Author Contributions

**Karen L. Pellegrin:** conceptualization, investigation, writing – original draft, methodology, writing – review and editing. **Tanner B. Barbour:** writing – review and editing, methodology, formal analysis, software, data curation. **Alicia J. Lozano:** data curation, software, formal analysis, methodology, project administration, writing – review and editing. **Alexandra L. Hanlon:** methodology, writing – review and editing, software, formal analysis, data curation, supervision. All authors have read and approved the final version of the manuscript.

## Conflicts of Interest

Karen L. Pellegrin discloses that she is a noncompensated member of the governing board of directors for the nonprofit Hawaiʻi Health Information Exchange.

## Transparency Statement

The lead author Karen L. Pellegrin affirms that this manuscript is an honest, accurate, and transparent account of the study being reported; that no important aspects of the study have been omitted; and that any discrepancies from the study as planned (and, if relevant, registered) have been explained.

## Data Availability

The data that support the findings of this study are available from Hawaiʻi Health Information Exchange upon reasonable request. Data are available from the author(s) with the permission of Hawaiʻi Health Information Exchange. Karen L. Pellegrin had full access to all of the data in this study and takes complete responsibility for the integrity of the data and the accuracy of the data analysis.
